# Huge Abdominal Mass in a Young Unmarried Lady - A Social and Psychotherapeutic Challenge

**DOI:** 10.7759/cureus.14672

**Published:** 2021-04-25

**Authors:** Naveed Ahmed, Taimoor A Khan, Muhammad M Saleem, Osama Bhatti, Muhammad A Zahid

**Affiliations:** 1 General and Pediatric Surgery, Combined Military Hospital, Quetta, PAK; 2 Internal Medicine, Headquarters Ghazaband Scouts Belleli, Quetta, PAK; 3 General and Pediatric Surgery, Combined Military Hospital, Peshawar, PAK; 4 General Practice, Monash University Health Services, Melbourne, AUS; 5 General Medicine, Army Medical College, Rawalpindi, PAK

**Keywords:** huge ovarian tumor, psychosomatic stress disorder, surface epithelial tumors, benign serous tumors

## Abstract

Large ovarian tumours are rare. Various diagnostic modalities are used to help in confirming the size and type of ovarian tumours. Laparotomy in a lateral decubitus position under general anaesthesia is the most common treatment approach. Here we present a case of a 17-year-old unmarried girl from whom a 13 kg ovarian tumor was resected. We highlight in this case report the mental stress that a patient goes through when accused of having an extramarital pregnancy by the community in a conservative society like ours. Furthermore, the importance of psychological support and counselling is paramount.

## Introduction

The presentation of large abdominal masses is rarely seen in clinical practice as early access to medical investigations and imaging is readily accessible [1]. This paper highlights a case report of a 17-year-old unmarried girl who presented with a four-month history of painless and growing abdominal distension and discusses the psychosocial implications of this presentation.

## Case presentation

A 17-year-old unmarried female presented to our resource-constrained clinic in a remote part of northern Pakistan, with a history of painless and progressive abdominal distension. Over the past four months, the patient’s abdomen had progressed to a diameter of 130 cm, restricting her ability to breathe and causing shortness of breath on exertion and on lying down. There was no significant personal or family history.

Socioeconomic factors such as lack of healthcare access and poor health literacy led to a delay in presentation. Also, it was important to acknowledge the sociocultural factors involved that affected the patient and her family. Community allegations of extramarital pregnancy had resulted in ostracization of the family and significant psychological distress to the patient, who appeared with a flat affect, depressed mood, poor eye contact, and reported frequent suicidal ideation due to the social pressure.

On examination, the patient was hemodynamically stable but dehydrated and pale. The abdomen was massively distended (130 cm), tense, non-tender, and prominent veins were seen on the anterior abdominal wall. However, the huge mass was palpable due to its sheer size the upper and lower limits could not be defined. Chest examination revealed symmetrical expansion with adequate air entry bilaterally, however, the patient was unable to take deep breaths because of likely splintage of the diaphragm.

Chest X-ray revealed rib crowding and elevation of the diaphragm to T5 (from its usual position of T8-T12). Abdominal ultrasound revealed a huge cystic and lobulated mass with internal septations occupying the whole abdominal cavity, most likely arising from the right ovary, along with a mild amount of fluid in the peritoneal cavity. An abdominal CT scan and CA-125 were also offered but rejected by the family due to cost restrictions as these investigations could not be performed locally.

Surgical excision was planned under regional anaesthesia due to the impact of the abdominal mass restricting respiration and lung capacity relatively contraindicating general anaesthesia. Incision extended from xiphisternum to symphysis pubis. A huge mass was seen originating from the right ovary with flimsy adhesions between mass and intestine/omentum which were easily freed. It was removed in total, sparing ipsilateral fallopian tube and ovary. Surrounding viscera were found to be uninvaded, which favoured the benign nature of the lesion. Furthermore, no metastases were observed in any organ. Due to pressure effects, the posterior aspect of the anterior abdominal wall was very hyperaemic. Two drains were placed in the peritoneal cavity that were removed on the third post-operative day. The patient’s recovery was uneventful, and she was discharged on the seventh post-operative day with suture removal and good wound healing observed on the 14th post-operative day. The patient and family were extremely satisfied and relieved. During pre-operative work, the father had mentioned several times that the patient had developed suicidal thoughts due to social stress. There has been no recurrence of tumour growth at four months follow-up.

**Figure 1:**
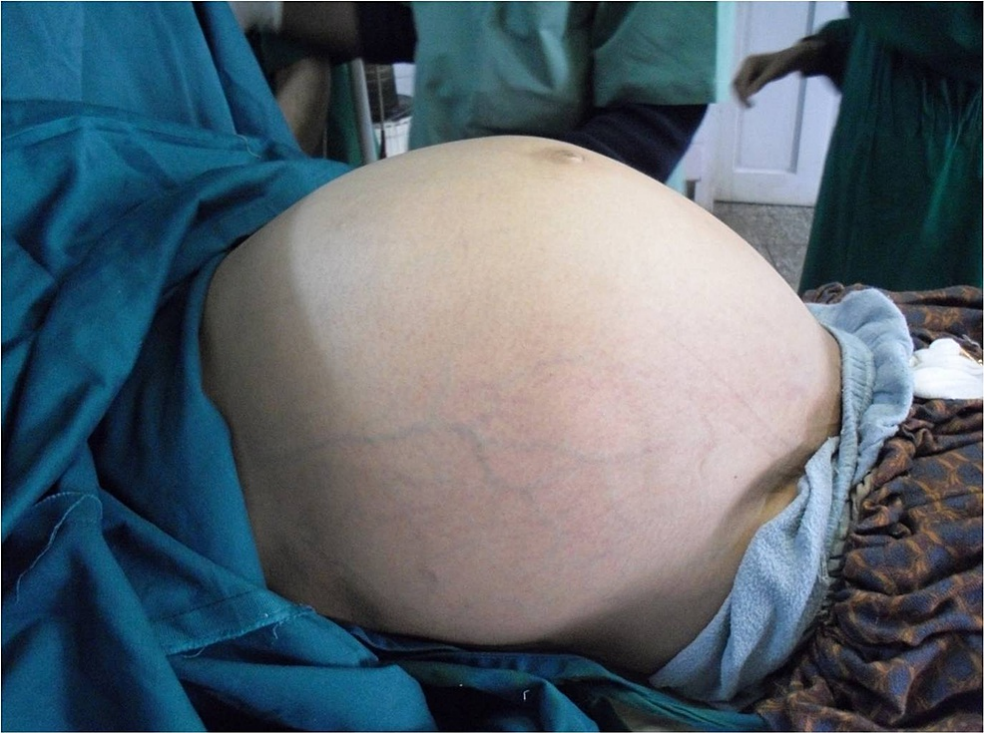
Massively distended abdomen (130 cm)

**Figure 2:**
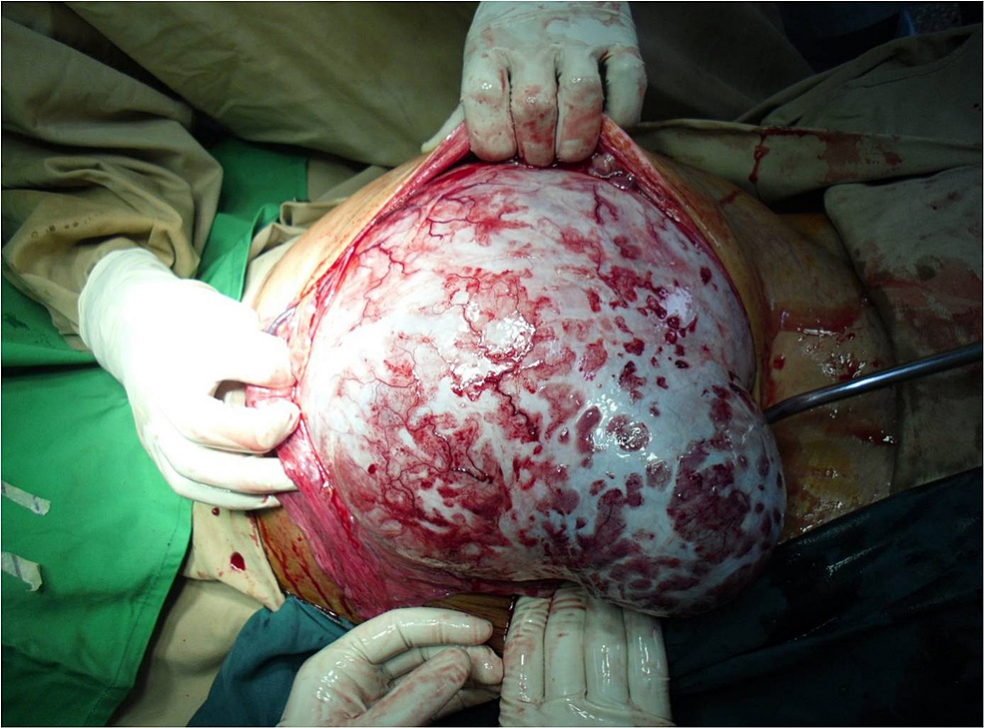
Huge mass originating from the right ovary

**Figure 3:**
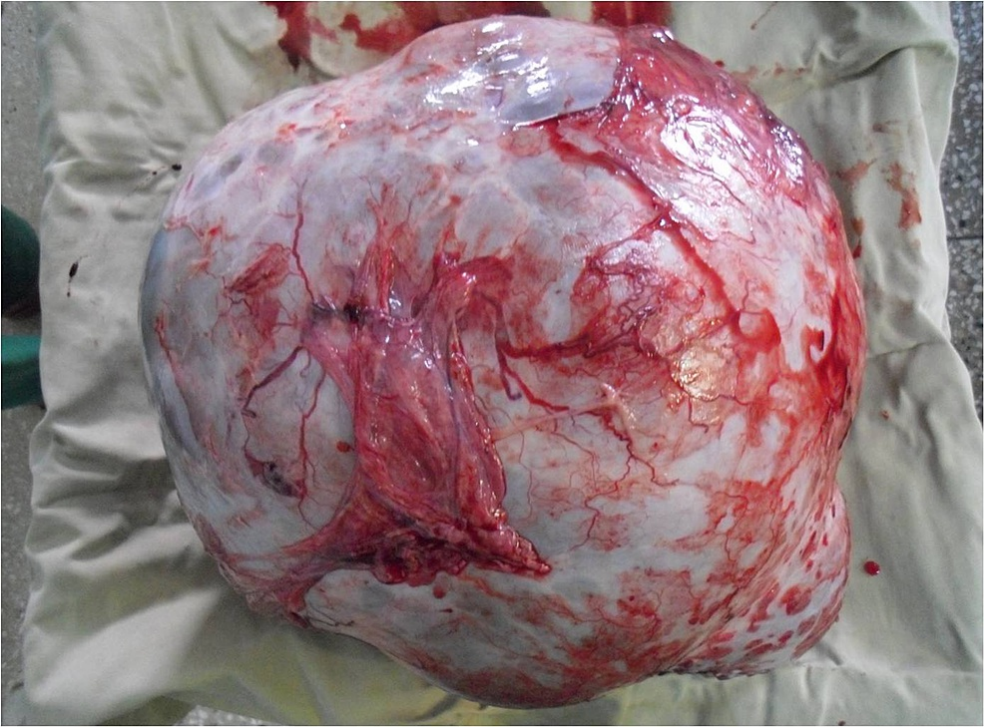
13 kg ovarian tumour

Macroscopic examination revealed a 13 kg specimen (30 cm x 30 cm x 26 cm), however, histopathology was refused by the family who instead requested to take the specimen home to demonstrate as evidence to their family and community members to prove that her abdominal distention was secondary to a tumour and not a foetus (Figures 1-3).

## Discussion

Some of the largest ovarian tumours in literature to date are a 148.6 kg mass as reported by Spohn in 1922 and a 79.4 kg mass as reported by Symmonds in 1963 [2]. Although ovarian tumours are relatively common [3], huge ovarian tumours are rare. This is in part due to the improvement of medical imaging and hence earlier detection of these tumours [4]. Moreover, earlier intervention results in earlier resection of these tumours and prevents them from growing to huge sizes. A variety of factors may contribute to the late presentation of the patient to the medical setup, such as financial constraints [4, 5], inaccessible medical facilities in rural areas, fear of finding a terminal diagnosis [3], and self-diagnoses of patients of getting obese [3]. However, our patient was accused of being pregnant out of wedlock; this social pressure prevented her from presenting to the hospital early, and so she was in an extremely fragile mental state.

Ovarian neoplasms are divided into germ cell, stromal, and surface epithelial. Surface epithelial tumours comprise 50% of all ovarian tumours and are categorised into benign and malignant types. Benign serous tumours include surface papillomas, adenofibromas, cystadenofibromas, and cystadenomas. Furthermore, grossly they are quite large, unilateral, and ovoid or spherical in shape [2], as seen in our patient. 

A variety of diagnostic approaches can be taken to diagnose the type of tumour. Ultrasonography [4] and computerized tomography (CT) scan [6] are the imaging modalities of choice, whilst raised tumour markers such as CA 125 [4] are strong predictors as well. Gross intraoperative examination may provide clues about the possible nature of the tumour, but histopathology is necessary for a conclusive diagnosis.

Various treatment approaches have been discussed in literature and usually include laparoscopy and laparotomy. Laparotomy under general anaesthesia is by far the most common approach taken [1-5, 7], with surgeons preferring the lateral decubitus position [5] to fight the effect of supine hypotension from the tumour compressing upon the inferior vena cava. However, our patient was operated on under regional anaesthesia in a supine position.

Furthermore, every case that we analysed had a total abdominal hysterectomy and salpingo-oophorectomy performed [1-4, 7], however, as our patient was young, it was decided to avoid this aggressive approach due to the benign intraoperative findings.

In addition to medical and surgical treatment, it is also important to assess the psychosocial impact of an ovarian tumour. Patients may report anxieties regarding their diagnosis, their appearance, the surgeries required, and the risks of malignant pathologies. Psychosomatic stress disorder related to ovarian tumours has been acknowledged only in two case reports [4, 5] and was treated only in one instance in the form of psychiatric rehabilitation [5]. The patient in our case report revealed significant anxiety and psychosocial stress due to the allegations of extramarital pregnancy from her community. This is of particular social importance in Pakistani society, where extramarital affairs are considered a major sin. According to the Pakistan Penal Code, adultery is a punishable crime [8]. Furthermore, in tribal and remote areas of Pakistan, elders of the tribe reportedly hand down various severe punishments to the accused in a local court (Jirga). All the stated physical factors coupled with the accusations of conceiving a child out of wedlock did take a toll on the mental health of our patient. As a result, she developed severe depression which eventually resulted in suicidal tendencies. She required extensive psychological support and counselling during her treatment.

## Conclusions

Ovarian tumours are relatively common, however, large sizes are rarely seen. Various diagnostic modalities are used to help in confirming the type of ovarian tumour. Laparotomy in a lateral decubitus position under general anaesthesia is the most common treatment approach. It is fundamental to consider the psychosocial impact of abdominal mass in conservative communities with limited health care access.
